# Frailty is associated with a history of falls among mobility-limited older adults—cross-sectional multivariate analysis from the BIOFRAIL study

**DOI:** 10.1007/s41999-025-01239-3

**Published:** 2025-05-27

**Authors:** Pernille Hansen, H. Nygaard, M. Schultz, F. Dela, P. Aagaard, Jesper Ryg, C. Suetta

**Affiliations:** 1 Geriatric Research Unit, Department of Medicine, Copenhagen University Hospital, Herlev and Gentofte, Gentofte, Denmark; 2 Geriatric Research Unit, Department of Geriatric and Palliative Medicine, Copenhagen University Hospital, Bispebjerg and Frederiksberg, Copenhagen, Denmark; 3 Faculty of Health and Medical Sciences, Institute of Clinical Medicine, CopenAge - Copenhagen Center for Clinical Age Research, University of Copenhagen, Copenhagen, Denmark; 4 Department of Emergency Medicine, Copenhagen University Hospital, Bispebjerg and Frederiksberg, Copenhagen, Denmark; 5 Department of Geriatrics, Copenhagen University Hospital, Hvidovre and Amager, Hvidovre, Denmark; 6 Department of Clinical Medicine, Faculty of Health, University of Copenhagen, Copenhagen, Denmark; 7 Xlab, Department of Biomedical Sciences, Faculty of Health and Medical Sciences, University of Copenhagen, Copenhagen, Denmark; 8 Laboratory of Sports and Nutrition Research, Riga Stradiņš University, Riga, Latvia; 9 Department of Sports Sciences and Clinical Biomechanics, University of Southern Denmark, Odense, Denmark; 10 Geriatric Research Unit, Department of Clinical Research, University of Southern Denmark, Odense, Denmark; 11 Department of Internal Medicine Geriatric Section, Copenhagen University Hospital, Herlev and Gentofte, Herlev, Denmark

**Keywords:** Falls, Muscle strength, Mechanical muscle function, Mobility-limited patients, Old age

## Abstract

**Key summary points:**

**Aim:**

To identify differences in characteristics between mobility-limited older adults with a history of falls and those at risk of falling but who have not yet fallen.

**Findings:**

Frailty and muscle strength were characteristics distinguishing between older adults with a history of falls and those absent of falls despite an increased risk of falling.

**Message:**

Frailty should be incorporated alongside handgrip strength (HGS) and sit-to-stand (STS) tests into routine evaluations of mobility-limited older adults referred for fall assessment.

**Abstract:**

**Purpose:**

We aimed to identify differences in characteristics between mobility-limited older adults with a history of falls and those at risk of falling, and to identify the parameter with the strongest predictive value on the risk of falling.

**Methods:**

Data included anthropometry, HGS, 30-s and 5-reps STS tests, maximal isometric knee extensor strength, gait speed (6 m), postural balance (tandem test), and muscle mass (BIA). Frailty was assessed using the Clinical Frailty Scale (CFS) and sarcopenia was evaluated according to the European Working Group on Sarcopenia in Older People 2 (EWGSOP2) guidelines. Outcomes of falls (past year), and depression (Geriatric Depression Scale 15) were self-reported.

**Results:**

Totally, 505 mobility-limited older adults (mean age 79.7 ± 6.3 years, 64.8% females) were included. Of these, 400 (79.2%) had experienced one or more falls within the past year (fallers), while 105 (20.8%) had not experienced a fall (at risk). Patients with experienced falls were more likely to feel depressed, had reduced handgrip strength, and reduced performance in both STS tests compared to those who had not fallen.

Frailty was the strongest individual parameter associated with a history of prior falls, even after adjusting for covariates such as depression and 30-s STS (aOR 3.80; 95% CI 1.70–8.50).

**Conclusions:**

Present study identified frailty as a key factor independently associated with a history of falls in this population. Additionally, handgrip strength and STS performance were key characteristics distinguishing between older adults with a history of falls within the past 12 months and those at risk of falling.

**Trial registration:**

NCT05795556

## Background

Falls among older adults are a major and increasing public health concern globally, as they constitute a leading cause of injury-related disability and mortality, with profound implications for this population [1, 2]. Annually, approximately 30% of community-dwelling individuals over the age of 65 are expected to experience at least one fall and this prevalence increases to nearly 40% among those aged 80 years and older [2–6]. The impact of falls extends beyond the acute physical injuries, often resulting in a phenomenon known as the "concern of falling" [7], which can trigger a cascade of negative outcomes, including reduced physical activity, social isolation, depression, further functional decline, and eventually, an increased likelihood of institutionalization [7, 8]. Indeed, falls have been reported to account for 53% of all injury-related deaths in older adults within the European Union, highlighting their significant impact in this population [9].

As the global population ages and the relative proportion of older adults continues to rise, the healthcare burden associated with fall-related injuries is expected to escalate substantially [10]. This underscores the need for effective and accurate assessment tools to reliably predict fall risk among older adults, in order to prevent falls, optimize healthcare resources, and improve quality of life for this population.

The etiology of falls is complex and multifactorial [11–16] with various factors implicated in increased fall risks, including declines in muscle strength, postural balance deficits, reduced gait speed, impaired cognitive function, and a history of previous falls [14, 16, 17]. However, these factors often interact in ways that can vary substantially between individuals, making it challenging to predict falls with high accuracy in the clinical setting.

Despite existing research, there is still no consensus on which specific parameters are most effective in distinguishing older mobility-limited adults at risk of falling from those who have already experienced a fall. While many studies have investigated differences between fallers and non-fallers, they often focus on only one or two factors at a time [5, 17–21], leaving a gap in the literature regarding the need for a more comprehensive assessment. Evaluating multiple parameters—such as muscle strength, muscle mass, balance, gait speed, and fall history—could provide a more holistic understanding of fall risk. Thus, to effectively predict fall risk, an assessment must first demonstrate its ability to distinguish older adults with a history of falls from those who are at risk but have not yet fallen.

Therefore, the aim of this study was to identify the difference in characteristics between mobility-limited older adults who have experienced one or more falls in the past year and those who are at risk of falling but have not yet fallen and to investigate the association between the parameter most strongly associated with the risk of falling.

## Material and methods

### Study design and setting

The Biofrail Study was designed as a cross-sectional cohort study involving patients referred to the geriatric outpatient fall clinic at Copenhagen University Hospital, Herlev and Gentofte, Denmark, between September 2021 and June 2023. All patients aged 65 years and older who were referred to the geriatric outpatient fall clinic were eligible for participation in the study. Exclusion criteria were severe dementia, communicative problems, and inability to walk independently without personal support (walking aids allowed).

All patients provided their written informed consent prior to enrolment in the study. The study received approval from the local Ethics Committee of Copenhagen and Frederiksberg (H-20057620) and the Danish Data Protection Agency (P-2021-619), and it was registered at ClinicalTrials.gov (NCT05795556). All experimental procedures were conducted in accordance with the Declaration of Helsinki.

### Assessments

To comprehensively assess the patients in the study, a range of objective and subjective measurements were conducted. These assessments aimed to evaluate key parameters related to muscle strength, functional capacity, muscle mass, and overall health status, providing a multidimensional perspective on the physical condition of the patients.

### Anthropometric measurements

Height (m) was measured without shoes to the nearest 0.1 cm and weight with light clothing to the nearest 0.1 kg. Subsequently, body mass index (BMI) was calculated as weight in kilograms divided by height in meters squared (kg/m^2^).

### Muscle strength and functional capacity

#### Handgrip strength

Handgrip strength (HGS) was assessed using a hand-held dynamometer (Jamar Smart, Sammons Preston Rolyan, Chicago, Illinois, USA) [18]. Patients were instructed to perform three trials with their dominant hand. If the highest value was recorded during the third trial, additional trials were conducted until no further increase in strength was observed, thereby determining the patients’ maximal HGS. The highest value was selected for subsequent analysis. To classify low HGS, cut-off values of < 27 kg and < 16 kg, for male and female patients, respectively, were applied, as defined by the European Working Group on Sarcopenia in Older People 2 (EWGSOP2) guidelines [19].

#### Maximal isometric knee extensor strength

The maximal isometric strength of the knee extensors (m. quadriceps femoris) was measured as the peak isometric knee extensor peak torque (Nm) during static MVC (maximum voluntary contraction) in a seated position using a belt-stabilized digital hand-held dynamometer (Lafayette, Model 01165) [20]. Patients were seated on an examination bed, and firmly strapped to the seat at the hip and just above the knees. The dynamometer was fixed with a strap on the distal front side of the tibia positioned just above (3 cm) the lateral malleolus, with the knee joint at 90° flexion (0° = full extension), and their feet not touching the floor. The lever arm distance from the application point of the dynamometer to the knee joint axis of rotation (lateral femoral condyle) was measured (m) and multiplied with the dynamometer force (N) to yield maximal voluntary contraction strength of the knee extensors expressed in torque units (Nm) (MVC). The highest peak torque of three attempts for each leg was selected for analysis.

#### Sit-to-stand performance

Sit-to-stand performance was evaluated using both the 30 s sit-to-stand (30-s -STS) test and the 5-repetition sit-to-stand (5-reps STS) test [21, 22]. The 30-s STS test measures the number of times a patient can rise from and sit down on a chair within a 30 s period [21, 23], while the 5-reps STS test measures the time it takes to complete five consecutive sit-to-stand cycles [22]. Low STS performance was defined as ≤ 8 repetitions on the 30-s STS test [24] and > 15 s on the 5-reps STS test [19].

#### Gait speed

Gait speed (GS) was measured at both habitual (self-chosen) and maximal speed over a 6 m course with patients allowed to use their usual walking aids [25]. GS was calculated by dividing the 6 m distance by the time it took the patients to complete the distance at both paces [25, 26]. Low GS was defined as ≤ 0.8 m/s [19].

#### Postural balance

Postural balance was measured using the tandem test [27], which involved three standing positions; (1) feet positioned side-by-side, (2) semi-tandem, heel of one foot beside the big toe of the other foot while maintaining parallel feet, and (3) tandem, one foot positioned directly in front of the other, heel touching toes. The patient was instructed to maintain each position for 10 s and the score was based on performance (seconds), with an overall maximum score of 30 [27]. Patients were allowed one practice attempt and one maximal attempt.

### Muscle mass

Muscle mass was measured using multi-frequency bioelectrical impedance analysis (BIA) (Inbody770 and InbodyS10; Biospace Co., Seoul, Korea) and reported as skeletal muscle index (SMI). However, since muscle mass measured with BIA tends to slightly overestimate muscle mass compared to dual-energy X-ray absorptiometry (DXA) [28–30], data were adjusted using the following equation [31]:

$$SMI \, = \, ALM*h^{ - 2} \left( {DEXA} \right) \, = \, 5.03 \, + \, 1.17 \, * \, ALM*h^{ - 2} \left( {BIA} \right) \, {-} \, 0.039* \, height$$

where appendicular lean mass (ALM) was the sum of muscle mass (lean mass) in the arms and legs, and height was expressed in meters (m). Low SMI was defined as < 7.0 kg/m^2^ and < 5.5 kg/m^2^, for males and females, respectively [19].

### Sarcopenia

In line with EWGSOP2 guideline [19], sarcopenia was identified when both low muscle strength and low muscle mass were observed. Muscle strength was considered low if patients showed reduced values in HGS, 30-s STS performance, or both. Severe sarcopenia was identified when a slow habitual gait speed was also present in addition to low muscle strength and mass [19].

### Frailty

Frailty was evaluated using the validated Danish version of the Clinical Frailty Scale (0–9) [32]. A score ≥ 5 specified the presence of frailty [33, 34].

### Patient reported outcome measures (PROMs)

The SARC-F questionnaire was employed as a screening tool to identify patients with probable sarcopenia [35]. Additionally, patients were asked to report any falls within the past year, and categorized into three groups: no falls, 1–3 falls, or 4 or more falls. For this study, patients were subsequently classified into two groups based on their responses: those who had experienced one or more falls (≥ 1 fall), categorized as fallers, and those who reported no falls, categorized as at risk of falling.

The Short Nutritional Assessment Questionnaire (SNAQ) was used to identify patients at risk of malnutrition [36]. Patients with a score < 2 were considered well-nourished, those with a score = 2 were classified as moderately malnourished, and those with a score > 2 indicated severe malnutrition.

To quantify the degree of depression, the Geriatric Depression Scale (GDS-15) was utilized [37–39]. A score of 0–4 was classified as normal, 5–8 suggested mild depression, 9–11 indicated moderate depression, and a score of ≥ 12 indicated severe depression [39, 40].

### Statistical analysis

Patients’ characteristics are presented for the total cohort and followed by differences between fallers and patients at risk of falling. All continuous data were visually inspected using residual plots and histograms to evaluate the normality of distribution and mean [± SD] or median (IQR) was used accordingly**.** Descriptive statistics were conducted to determine the relative frequency (%), while independent *t*-tests, Mann–Whitney U, and chi-squared tests were performed to investigate potential group differences.

Frailty, defined as CFS ≥ 5, demonstrated the strongest statistical significance in the initial descriptive analyses. Consequently, both a crude and a multivariable regression model were applied to examine the association between frailty and fall history, presenting odds ratio (OR) with 95% confidence intervals. The multivariable model adjustments (aOR) were performed by forward selection of covariates that differed statistically between the group of patients at risk of falling and the group of patients with a history of falls, with adjustments made for GDS-15 and the 30-s STS test. The covariates, which were included in the models are specified in the tables, as they occur in the adjusted model.

Statistical significance was set at p ≤ 0.05 (2-tailed). All statistical analysis was performed in SAS Studio.

## Results

A total of 505 patients were included in the study (mean age 79.7 ± 6.3 years, 64.8% females). The characteristics of the study cohort are presented in Table 1. Of these, 400 (79.2%) had experienced one or more falls within the past year (fallers), while 105 (20.8%) had not experienced a fall (at risk). There were no differences in the distribution of sex, age, BMI, or the prevalence of malnutrition between the two groups. Mild-to-severe depression was present in 30.4% of the total cohort. Patients who had experienced one or more falls were more likely to feel depressed than those who had not fallen within the last 12 months (fallers: 32.8% vs. at risk: 20.8%, p = 0.023).

**Table 1:** Patient characteristics presented for the total group and according to fall status. The p-value reflects the level of statistical significance between the group of fall patients and patients at risk of falls for each variable

Characteristics	All patients (n = 505)	Fall patients (n = 400)	Risk of fall (n = 105)	p-value
Sex				0.109
Male, n (%)	178 (35.2)	134 (33.5)	44 (41.9)	
Female, n (%)	327 (64.8)	266 (66.5)	61 (58.1)	
^α^Age	79.7 ± 6.3	79.8 ± 6.5	79.4 ± 5.4	0.519
^β^BMI	25.3 [22.8–28.5]	25.3 [22.7–28.5]	24.9 [23.0–28.3]	0.676
SARC-F High SARC-F, n (%)	3 [2–6] 253 (50.1)			
High SNAQ^a^, n (%)	84 (16.7)	71 (17.9)	13 (12.4)	0.179
High GDS-15^b^, n (%)	145 (30.4)	125 (32.8)	20 (20.8)	0.023*
^α^SMI^c^ Low SMI, n (%)	6.6 ± 1.1 123 (26.1)	6.6 ± 1.2 95 (25.5)	6.7 ± 1.1 28 (28.3)	0.653 0.571
Low SMI, n (%) Males Females	50 (30.5) 73 (23.7)	39 (31.7) 56 (22.4)	11 (26.8) 17 (29.3)	0.557 0.265
^β^Handgrip strength^d^ (kg) Low HGS, n (%)	21.7 [17.1–28.9] 131 (27.0)	21.6 [16.8–27.9] 111 (28.9)	22.7 [18.3–31.6] 20 (19.6)	0.027* 0.060
Low HGS, n (%)				
Males	43 (25.3)	35 (27.6)	8 (18.6)	0.243
Females	88 (27.8)	76 (29.6)	12 (20.3)	0.154
^β^30-sec STS^e^ Low 30-s STS, n (%)	10.0 [7.0–13.0] 159 (36.9)	10.0 [6.0–13.0] 132 (39.6)	11.0 [8.0–14.0] 27 (27.6)	0.009* 0.029*
^β^5-reps STS^f^ (sec) Low 5-reps STS, n (%)	12.7 [10.1–16.2] 135 (31.0)	12.9 [10.4–16.5] 111 (32.7)	11.5 [9.2–15.1] 24 (24.7)	0.006* 0.133
^α^GS Hab^g^ (m/s) Low GS, n (%)	0.87 ± 0.25 183 (40.6)	0.86 ± 0.25 146 (41.4)	0.90 ± 0.25 37 (37.8)	0.056 0.520
^α^GS Max^g^ (m/s) Low GS, n (%)	1.16 ± 0.36 72 (16.0)	1.15 ± 0.36 59 (16.8)	1.20 ± 0.36 13 (13.3)	0.085 0.404
^β^MVC KE torque Right^h^ (Nm/kg)	0.88 [0.69–1.15]	0.88 [0.69–1.14]	0.89 [0.70–1.16]	0.635
^β^MVC KE torque Left^h^ (Nm/kg)	0.76 [0.59–1.02]	0.76 [0.57–1.01]	0.80 [0.64–1.06]	0.110
^β^Tandem^i^ (sec)	25.2 [19.9–30.0]	25.0 [19.5–30.0]	26.5 [20.0–30.0]	0.203
^α^Frailty^j^ CFS ≥ 5, n (%)	4.00 [3.00–5.00] 121 (26.1)	4.00 [3.00–5.00] 112 (30.9)	3.00 [3.00–4.00] 9 (8.9)	< 0.001* < 0.001*
Sarcopenia^k^, n (%)	57 (14.2)	47 (15.2)	10 (11)	0.313

Descriptive statistics: mean ± SD; median [Q1-Q3]. Differences in proportions between groups (fall vs at risk) were investigated using chi-square tests (Pearson Chi-Square). High SARC-F was defined as a score ≥ 4. Low HGS defined as values < 27 kg and < 16 kg, for males and females, respectively. Low 30-s STS was defined as values ≤ 8 repetitions and low 5-reps STS was defined as < 15 s. Low SMI was defined as values < 7.0 kg/m^2^ and < 5.5 kg/m^2^, for males and females, respectively. Low GS was defined as values ≤ 0.8 m/s (see text for details)

*30-s STS* 30 s sit-to-stand, *5-reps STS* 5 repetitions sit-to-stand, *BMI* Body Mass Index, *CFS* Clinical Frailty Scale, *GDS-15* the Geriatric Depression Scale, *GS Hab* Habitual gait speed, *GS Max* Maximal gait speed, *HGS* Handgrip strength, *MVC KE* torque Maximal isometric Knee Extensor torque, *SMI* Skeletal Muscle Index, *SNAQ* the Short Nutritional Assessment Questionnaire, *STS* sit-to-stand

*Significant difference (*p* < 0.05) between patients who had experienced one or more falls and patients who had not fallen within the last 12 months

^a^n = 502 (fall n = 397, risk n = 105)

^b^n = 477 (fall n = 381, risk n = 96)

^c^n = 472 (fall n = 373, risk n = 99)

^d^n = 486 (fall n = 384, risk n = 102)

^e^n = 431 (fall n = 333, risk n = 98)

^f^n = 436 (fall n = 339, risk n = 97)

^g^n = 451 (fall n = 353, risk n = 98)

^h^n = 420 (fall n = 328, risk n = 92)

^i^n = 475 (fall n = 372, risk n = 103)

^j^n = 464 (fall n = 363, risk n = 101)

^k^n = 401 (fall n = 310, risk n = 91)

^α^Independent Sample T-test

^β^Mann-Whitney U test

### Muscle mass and physical capacity

Low SMI emerged in 26.1% of the patients, with no differences between the groups.

A significant difference was observed in HGS (median [IQR]) with 21.6 [16.8–27.9] kg in fallers and 22.7 [18.3–31.6] kg in patients at risk (p = 0.027), however, there was no difference between the presence of low HGS in the two groups (fallers: 28.9% vs. at risk: 19.6%, p = 0.060).

Patients at risk of falling performed better than those who had experienced a fall in the 30-s STS (at risk: 11 [8.0–14.0] reps vs. fallers: 10 [6.0–13.0] reps, p = 0.009), as well as in the 5-reps STS test (at risk: 11.5 [9.2–15.1] sec vs. fallers: 12.9 [10.4–16.5] sec, p = 0.006). Likewise, the prevalence of reduced 30-s STS performance was higher in fallers (39.6%) than those at risk of falling (27.6%) (p = 0.029). No differences in the prevalence of reduced 5-reps STS performance were observed between the two groups (p = 0.133).

There was a trend but not a significant difference in habitual GS (mean ± SD) between fallers (0.86 ± 0.25 m/s) and those at risk of falling (0.90 ± 0.25 m/s) (p = 0.056), with a similar tendency (p = 0.085) observed for maximal GS (fallers: 1.15 ± 0.36 m/s vs. at risk: 1.20 ± 0.36 m/s).

Likewise, no differences were observed in maximal isometric knee extensor strength in either right (fallers: 0.88 [0.69–1.14] Nm/kg vs. at risk: 0.89 [0.70–1.16] Nm/kg, p = 0.635) or left (fallers: 0.76 [0.57–1.01] Nm/kg vs. at risk: 0.80 [0.64–1.06] Nm/kg, p = 0.110) leg between the groups.

Additionally, no difference was observed between the groups in their ability to maintain postural balance, as measured by the tandem (fallers: 25.0 [19.5–30.0] sec vs. at risk: 26.5 [20.0–30.0] sec, p = 0.203).

### Frailty and sarcopenia

More than 25% (n = 121) of the patients were found to be frail (CFS ≥ 5), with a higher proportion in the patients who had experienced one or more falls, than those at risk of falling, 30.9% (n = 112) and 8.9% (n = 9), respectively (p < 0.001).

The prevalence of sarcopenia was 14.2%, with no differences between the groups (fallers: 15.2% vs. at risk: 11.0%, p = 0.313).

### The association between frailty level and the history of falling

After identifying frailty as the most discriminating variable, its association with the risk of falling was further investigated. A total of 381 patients with complete records for the GDS-15, 30-s STS, and CFS were included in this analysis. In the crude model, frailty was significantly associated with falls, with an Odds Ratio (OR) of 4.19 (95% CI 1.94–9.04). After adjusting for depression and the 30-s STS, aOR^b^ was found to be 3.98 (95% CI 1.76–9.01) (Table 2).

**Table 2:** Results from the multivariable logistic regression analysis investigation of the association between frailty and history of falling

	Crude OR (95% CI)	aOR^a^ (95% CI)	aOR^b^ (95% CI)
Frailty	4.19 (1.94–9.04)	3.90 (1.80–8.49)	3.80 (1.70–8.50)

*CI* confidence interval, *OR* odds ratio.

^a^Model adjusted for depression

^b^Model adjusted for depression and 30-s STS

## Discussion

In the present study, we focused on identifying key characteristics that could distinguish older adults who have experienced one or more falls in the past year from those at risk of falling but have not yet fallen. We aimed to take a broader approach by simultaneously assessing multiple parameters, including muscle strength, muscle mass, postural balance, gait speed, and fall history. By adopting such a comprehensive approach, we aimed to identify the parameters most strongly associated with fall risk and their potential utility in predictive models. Our analysis revealed that frailty assessed by the CFS was the single parameter most strongly associated with a history of prior falls in mobility-limited older adults, also after adjusting for covariates such as depression and 30-s STS. Additionally, we found that maximal muscle strength, measured through HGS and STS, was the characteristic that could distinguish between older adults who have experienced falls in the past year and those at risk of falling, and should be considered in future models to predict fall risk in this population.

### Frailty

The interplay between falls and frailty is closely intertwined, as both are sensitive markers of declining physical health in older adults [41]. Falls are often seen as a key consequence of frailty because of the loss in lower limb muscle strength, postural balance, and inter-muscular coordination, which are some of the physical hallmarks of frailty [42, 43], altogether increasing the likelihood of falling.

The present findings that older individuals who have experienced at least one fall in the past year are more likely to be frail than those who are at risk of falling yet managing to abstain from falls are supported by previous reports demonstrating an association between frailty and falls [44–47]. In a systemic review, Fhon et al. [45] demonstrated an odds ratio between fall incidence and frailty syndrome of 1.80 (95% CI 1.51–2.13), in line with a meta-analysis from Cheng et al. [46], who showed that the risk of falls in frail older adults was higher than in their more robust peers (OR 2.50; 95% CI 1.58–3.96).

One possible explanation for frail older people being more prone to falls is their compromised ability to recover from acute balance disturbances or more chronic physical challenges, respectively. Frailty is associated, among other things, with reduced muscle strength and mass (sarcopenia), slower gait speed, impaired balance, and reduced coordination [42, 43]—factors that are all critical for maintaining adequate postural control and avoiding falls [16]. Once a fall occurs, frail individuals also face a higher risk of musculoskeletal injuries, including hip and thigh fractures, further exacerbating their frailty and creating a vicious cycle [41].

Moreover, frailty is not just a physical condition but also includes cognitive decline and is often accompanied by signs of malnutrition, and general functional impairments [42, 48], all of which can contribute to an increased risk of falling. In contrast, it can be speculated that those at risk of falling but not yet frail (non-frail non-fallers) still possess some resilience and compensatory mechanisms that may help to maintain adequate control of postural balance and prevent falls, despite sharing similar risk factors, such as reduced muscle mass and gait speed.

### Muscle strength

Daneshjoo et al. [49] observed greater quadriceps and hamstrings strength in non-fallers compared to fallers, as well as reduced bilateral asymmetry in non-fallers, in alignment with earlier reports from Bartosch et al. [41] and Yang et al. [50]. These authors suggest that increased levels of strength in weight-bearing muscles may play a critical role in reducing fall risk by preserving postural control and mobility. However, contrary to these, our findings did not reveal any differences in maximal isometric knee extensor strength between those who have experienced at least one fall in the past year and those who are at risk of falling.

The lack of group difference in lower limb muscle strength in the present cohort suggests that factors other than maximal isometric strength are decisive for the elevated risk of falls such as reduced levels of maximal muscle power, rapid force capacity (rate of force development), muscle endurance, coordination, or neuromuscular efficiency, which all are factors not captured in isometric strength measurements. In fact, the present study observed differences in sit-to-stand (STS) performance, including both the 30-s STS and 5-reps STS tests, between those who had experienced a fall and those who had avoided falls but still were at risk of falling. Unlike isometric knee extension tests that primarily assess isolated muscle strength, STS tests place greater demands on these functional capacities by requiring the integration of multiple systems, including dynamic strength, postural control, and muscle endurance. This multifaceted nature makes STS tests potentially more sensitive in distinguishing between fallers and non-fallers.

Further, both the 30-s and the 5-reps STS tests have been validated as reliable measures of lower limb muscle power [51–54], which is a critical determinant of mobility and functional independence in older adults. The ability to generate power rapidly is essential for tasks such as recovering balance after a perturbation—which are highly relevant in fall prevention. Given the dynamic nature of STS tests, the observed differences in STS performance likely reflect impairments in muscle power generation among fallers. This finding suggests that muscle function and/or muscle power, rather than isometric strength alone, may be a crucial factor in distinguishing fallers from those at risk of falling [55].

Therefore, incorporating STS tests into fall risk assessments may provide a more comprehensive evaluation of functional ability, as these tests better replicate the real-world functional challenges faced by older adults compared to isolated strength measurements.

### Gait and postural balance

Multiple reviews have repeatedly highlighted gait and balance disorders as some of the most significant risk factors disposing for falls in older people [11, 16, 56]. An umbrella review by Jepsen et al. (2022) concluded that gait speed was the only test showing moderate evidence in predicting falls [57]. Surprisingly, the present study did not reveal any significant differences in either habitual or maximal gait speed between older individuals who had experienced falls in the past year and those at risk of falling. We did find a difference in habitual gait speed that was very close to being statistically significant (p = 0.056), suggesting that the lack of significance may be due to insufficient statistical power to detect the difference. However, the actual difference between the two groups was only 0.06 m/s, which is below the threshold for the minimal clinically important difference. This raises the question of whether the observed difference, despite being statistically close, is clinically meaningful. Thus, these findings suggest that while gait speed may be an important factor in fall risk, the practical significance of small differences in gait speed might be limited in this particular population. Similarly, no differences were found in postural balance performance measured by the tandem test, a widely used assessment in fall clinics [58]. One possible explanation for the lack of differences could be the characteristics of our study population. Since all participants were referred to the outpatient clinic for fall assessment, they may already share certain clinical risk factors, potentially masking any differences in gait speed or balance performance between fallers and those at risk of falling. This referral-based selection could have reduced the distinctions that might otherwise be seen in a more heterogeneous population.

Nevertheless, the present findings raise questions about the utility of such tests for accurately identifying individuals at risk of falls and it may be necessary to employ more specific and sensitive balance assessments to detect subtle impairments. In support of this notion, previous studies [59, 60] have shown that the tandem test, when performed on a force platform, can reveal differences in various sway parameters between fallers and non-fallers, indicating its potential to detect balance deficits that might not be captured by standard clinical assessments.

### Limitations and strengths

One of the main limitations of this study was the composition of our study population. Since all patients were referred for fall assessment, they were already identified as being at risk for falls, which may have reduced the ability to detect significant differences between fallers and non-fallers. This referral bias could have reduced distinctions in key variables such as gait speed and balance performance, making it more difficult to observe the differences reported in more heterogeneous populations. Furthermore, the cross-sectional design of the study limits the ability to establish causal relationships between the observed variables and fall risk. In particular, frailty was assessed after the fall event(s) had occurred, which means it cannot be determined whether frailty was a predisposing factor per se or a consequence of falling. Although it is plausible that similar associations would be observed using longitudinal study designs, only a prospective design would allow to establish the temporal relationship between frailty and falls. Additionally, the dependence on retrospective self-reported prior falls introduces the potential for recall bias. Hence, it would be interesting to reproduce the current study in a population of community-dwelling older adults within a longitudinal framework to better explore causality and minimize recall bias.

A strength of this study was the comprehensive assessment battery used. Thus, data collection comprised a wide range of risk factors, including malnutrition, depression, muscle mass, muscle strength, physical function, and balance. This holistic approach allowed to thoroughly evaluate the interplay between these variables and determine which characteristics could effectively distinguish between individuals who had experienced one or more falls within the past 12 months and those absent of falls yet at elevated risk of falling. By including multiple domains of frailty and function, the present data provide valuable insights into the multifactorial nature of fall risk in mobility-limited older adults.

### Clinical implications and perspectives

Preventing falls is important to reduce fall-related injuries and hospitalizations, ultimately enhancing the quality of life and independence for older adults and leading to significant cost savings for healthcare systems. Establishing effective screening tools is essential to achieve this goal. The present findings highlight the importance of incorporating frailty assessments alongside measures of muscle strength, such as HGS and STS tests, into routine evaluations of mobility-limited older adults referred for fall assessment. The observation that individuals who have experienced at least one fall in the past year are significantly more frail than those who are at risk of falling suggests that frailty may represent a critical threshold that, once crossed, dramatically increases fall risk. Given the strong association between frailty and fall history, as well as the ability of muscle strength measures to distinguish fallers from those at risk, clinicians should consider integrating these parameters into fall risk prediction models.

Early identification of individuals at elevated risk of falls may allow for targeted prophylactic intervention to be performed, potentially reducing the incidence of falls and their associated consequences, hence potentially leading to improved patient outcomes.

## Conclusion

This study of mobility-limited older adults referred for fall assessment showed that frailty emerged as a pivotal factor associated with a history of falls, also after adjusting for relevant confounders. Additionally, handgrip strength and STS performance were key characteristics distinguishing between individuals who had experienced one or more falls within the past 12 months and those absent of falls despite of increased risk of falling. The present findings highlight the importance of addressing frailty and maintaining muscle function in fall prevention strategies, particularly in clinical settings where older adults are already identified as being at increased risk of falls. However, as frailty was assessed after the fall events, any direct causality cannot be inferred from the present observations. Future longitudinal studies are needed to determine whether frailty primarily acts as a risk factor or if it may also develop as a consequence of falls.

## Data Availability

The data used in the present study are available from the corresponding author [PH] upon reasonable request.
